# Reframing cigarettes as social currency: A randomized survey experiment on the role of warning images and pricing

**DOI:** 10.18332/tid/211071

**Published:** 2025-11-06

**Authors:** Yabin Xing, Wei Wen, Gang Wang, Kecheng Du

**Affiliations:** 1 Department of Physical Education, Wuhan University, Wuhan, China; 2 Tongji Hospital affiliated to Tongji Medical College of Huazhong University of Science and Technology, Wuhan, China; 3 Merchants' Guild Economics and Cultural Intelligent Computing Laboratory of Ningbo University, Ningbo, China; 4 Institute of Modern Communication Studies, School of Humanities and Communication, Ningbo University, Ningbo, China; 5 School of Journalism and Communication, Wuhan University, Wuhan, China

**Keywords:** social currency, warning images, cigarette price, social intimacy

## Abstract

**INTRODUCTION:**

In China, cigarettes function as both consumer goods and ‘social currency’. Despite increased awareness of smoking risks, cigarette gifting persists. This study examines whether graphic warning images and price increases can reduce the social value of cigarettes in gifting contexts.

**METHODS:**

A survey experiment was conducted (n=744), randomly assigning participants to a control group (n=189), a price treatment group (n=285), and an image warning group (n=270). Eligible participants were adults with stable incomes. Participants in the price treatment group viewed the same cigarette brands as in the control condition but with retail prices doubled relative to the market price, whereas those in the image treatment group viewed cigarette packs with added graphic warning images while prices remained unchanged. The primary outcomes were willingness to gift or receive cigarettes in strong and weak relationships (1=very unwilling to 5=very willing) and brand tier preference (1=low, 2=mid, 3=high), and logistic regressions were applied to assess treatment effects. All comparisons were made against the control group, and logistic regression results are presented as coefficients (β) with 95% confidence intervals (CI).

**RESULTS:**

Graphic warning images significantly reduced willingness to gift cigarettes (β= -0.88; 95% CI: -1.33 – -0.42, p<0.001) and expectations to receive cigarettes (β= -0.62; 95% CI: -1.08 – -0.16, p<0.01) in weak relationships but had no significant effect in strong relationships. Price increases did not affect gifting willingness but reduced brand preference in weak relationships (β= -0.67; 95% CI: -1.11 – -0.23, p<0.01).

**CONCLUSIONS:**

Graphic warnings effectively weaken the symbolic value of cigarettes in non-intimate relationships, while price increases alone are insufficient. Tobacco control strategies should prioritize altering symbolic meanings rather than relying solely on economic measures.

## INTRODUCTION

In China, cigarettes are not just personal consumer goods but also function as a ‘social currency’ for building and maintaining relationships^1^. Strict government regulation keeps cigarette prices stable and transparent. Their liquidity and monetary attributes are further enhanced by vendors’ willingness to repurchase them. The wide price range of cigarettes (5–100 RMB per pack, see Supplementary file Notes) makes mid-to-high-end brands symbols of socio-economic status in consumption, sharing, and gifting. Meanwhile, the festive and symbolic design of cigarette packaging further enhances their marginal value in social settings. Vibrant colors, patterns of good fortune, and traditional cultural symbols on packaging add to the symbolic value of cigarettes, making mid-to-high-end brands popular social gifts, even for non-smokers^2^.

Despite health campaigns increasing public awareness of smoking’s harms, the social function of cigarettes persists, with the gifting of mid-to-high-end brands growing in recent years^3,4^. This phenomenon starkly contradicts the assumption of traditional tobacco control research, which posit that increased public awareness of smoking-related harms should lead to reduced cigarette consumption and social usage. The social gifting of cigarettes significantly increases their circulation and consumption within society, exposing both smokers and non-smokers to cigarettes and thereby undermining the effectiveness of tobacco control measures^5^. Thus, curtailing the social function of cigarettes, beyond merely discouraging smoking, remains a major challenge in tobacco control.

Efforts to curb the social gifting of cigarettes have primarily focused on three strategies: enhancing health awareness, altering cigarette packaging, and using price levers to suppress consumption.

Firstly, increasing public awareness about the risks of smoking is widely regarded as a key strategy for reducing smoking and cigarette gifting. Research suggests that raising public awareness fosters intentions to quit smoking and, in theory, should reduce cigarette gifting as well^5-8^. This model has been effective in North America and Europe, where health education campaigns in the 1960s significantly reduced smoking and cigarette gifting practices^7,9^. In contrast, in China, while public awareness of the harms of smoking has risen sharply over the past two decades, with 78% of smokers acknowledging the link between smoking and lung cancer, smoking rates have not declined^3,10^. Moreover, the consumption of mid-to-high-end cigarettes for gifting purposes has surged, indicating that health awareness alone does not necessarily curb cigarette gifting^3,10^. This phenomenon suggests that social relationships and cultural factors may play a larger role in cigarette gifting behaviors than previously assumed.

Secondly, health warning messages on cigarette packaging are another widely adopted measure. These warnings, which often include both textual and graphic elements, are intended to raise awareness of the health risks associated with smoking and reduce both consumption and gifting^8,11^. While this approach has been successful in many countries, its impact in China has been limited. In China, textual warnings on cigarette packs have not significantly deterred cigarette gifting, as the effect appears to be more about individual intention rather than actual behavioral change^8^. While individuals may express an intention to reduce gifting, this does not always translate into actual reductions in behavior^8,12^. Moreover, the cultural significance of cigarettes as social gifts, particularly during festivals and family gatherings, complicates the effectiveness of health warnings. The debate continues about whether packaging changes could disrupt these cultural norms, but empirical evidence remains sparse.

Thirdly, raising cigarette retail prices is considered one of the most effective ways to reduce smoking prevalence^13^. However, this strategy has had a more ambiguous impact on cigarette gifting, particularly in China. Despite several rounds of price hikes, cigarette consumption remains stable, and the consumption of premium cigarette brands as gifts continues to grow^1^. In China, premium cigarettes like ‘Zhonghua’ symbolize social status and respect^9,14^. And price increases often lead consumers to switch to lower tier brands rather than reduce overall consumption^15^. This behavior highlights the low price elasticity of cigarettes, where consumers tend to substitute premium brands with cheaper alternatives rather than cut back on gifting altogether^16,17^.

Lastly, prior research on social networks has highlighted the distinction between strong and weak relationships, where strong relationships refer to close and emotionally intensive relationships such as family and close friends, and weak relationships refer to more casual or instrumental relationships such as acquaintances or colleagues^18^. This distinction is particularly relevant in the context of cigarette gifting, as cultural norms may shape different expectations of gifting and receiving behavior depending on the relational closeness. In this study, we therefore examine both strong and weak relationship contexts to capture these variations in social exchange.

This study examines how warning images and pricing affect the social role of cigarettes as gifts. A survey experiment is employed to address the following questions: ‘How do health warning images alter the value of cigarettes as social gifts and influence individual social behaviors?’, and ‘Can price changes effectively curb the circulation of cigarettes in social contexts?’.

## METHODS

This study, approved by the ethics committee of Wuhan University (Approval Number: WHU-HSS-IRB2024021), adopts a survey experiment method, which combines the advantages of both surveys and experiments. Participants were allocated to a control group, a price treatment group, or a warning label treatment group.

### Participants

The experiment was conducted in June 2024 at a university in Wuhan, where 60 students were randomly selected as the starting point. From there, the sample was expanded to include social members with stable incomes through their social networks. Eligibility criteria required participants to be adults (aged ≥18 years), non-institutionalized, and capable of completing the questionnaire independently. The final sample was divided into three groups: the control group, the price treatment group, and the warning label treatment group, with a total sample size of 744 participants (Supplementary file Table S1). Priori power calculations were conducted based on expected effect sizes from previous studies of tobacco warning interventions, suggesting that a sample of approximately 600 participants would be sufficient to detect medium effects (power=0.80, α=0.05). Our final sample size of 744 therefore provided adequate statistical power.

### Study design

The randomization was implemented at the time of recruitment; each recruited student was randomly allocated to a group and received the corresponding version of the questionnaire. The control group was shown the packaging and current prices of three cigarette brands (Yuxi, Zhonghua, and Huanghelou 1916), with health warning information presented in text form, serving as the baseline to study the effects of other variables (n=189)^2^. The price treatment group simulated an extreme scenario by doubling the price of cigarettes (n=285). The warning label treatment group used packaging with added warning images, keeping the price unchanged (n=270). The questionnaires were distributed by trained research assistants following a standardized protocol. Data collection was conducted once without follow-up. Blinding was not feasible given the nature of the intervention, as participants could clearly see the group-specific materials. The sample size of the first group was slightly smaller than the other two, possibly because the scenario presented in the first group’s survey was consistent with real-life situations, while the second and third groups created entirely new scenarios that intrigued the respondents, leading to a higher response rate overall.

### Measurement

The study distinguished between willingness to gift and willingness to receive cigarettes in strong and weak relationship contexts. For gifting, participants were asked: ‘Would you gift cigarettes to friends or family on a holiday?’ (strong relationship) and ‘Would you gift cigarettes to someone to thank them for a favor?’ (weak relationship). For receiving, participants were asked: ‘Would you accept cigarettes from friends or family as a holiday gift?’ (strong relationship) and ‘If someone wanted to thank you for a favor with a gift of cigarettes, would you accept it?’ (weak relationship). All responses were measured on a 5-point Likert scale (1=very unwilling, 2=unwilling, 3=neutral, 4=willing, 5=very willing).

To assess brand tier preference, respondents who expressed willingness to gift or receive cigarettes were asked about the expected brand tier. Specifically, brand choices were categorized into three tiers: 1=Yuxi (low-tier), 2=Zhonghua (mid-tier), and 3=Huanghelou 1916 (high-tier). Participants were presented with the following questions: ‘What brand of cigarettes would you choose to gift to friends or family for a holiday?’, ‘What brand of cigarettes would you expect to receive from friends or family for a holiday?’, ‘What brand of cigarettes would you choose to gift to someone to thank them for a favor?’, and ‘What brand of cigarettes would you expect to receive from someone for a favor?’. Responses were subsequently recoded into the three-tier classification for analysis.

Control variables included demographic and socioeconomic characteristics: age, gender (0=female, 1=male), urban/rural residence (0=rural, 1=urban), marital status (0=single, 1=married or cohabiting), children (0=none, 1=at least one child), education level (1=primary school, 2=middle school, 3=college diploma, 4=Bachelor’s degree, 5=postgraduate), and household income (log-transformed for regression analysis). Two additional control variables were used: 1) the number of smokers in the respondent’s social circle, measured by ‘How many people around you are smokers?’ (responses: 1=very few to 5=very many); and 2) smoking hazard perception, measured at pre-test and post-test, based on the question ‘How harmful is smoking to smokers?’ (responses: 1=very small to 5=very large). The change in risk perception was calculated as the difference between pre-test and post-test scores. All demographic and behavioral covariates were considered potential confounders and were included as control variables in regression models.

### Statistical analysis

Descriptive statistics were first calculated to summarize participant characteristics and outcome variables, including means and standard deviations for continuous variables and proportions for categorical variables. Independent-samples t-tests were used to compare willingness to gift and receive cigarettes between groups.

To examine intervention effects, ordered logistic regression models were employed for brand tier preferences, while binary logistic regression models were used for willingness to gift and receive cigarettes. All models included demographic and socioeconomic covariates (age, gender, urban/rural residence, marital status, children, education level, and log-transformed household income), as well as two behavioral covariates: number of smokers in the respondent’s social circle and perception of smoking hazards (pre-test and change scores). These covariates were considered potential confounders and included as adjustment variables.

Missing values were minimal (<5%) and complete data were available for 744 participants, with cases containing missing values excluded via listwise deletion. All statistical analyses were performed using Stata 17.0. Statistical significance was set at p<0.05. Results are reported with exact p-values where available, and thresholds of p<0.05, p<0.01, and p<0.001 are indicated to denote different levels of significance.

## RESULTS

### Descriptive statistical analysis

Descriptive statistics for the study sample are presented in Table 1. Overall, 59% of respondents were willing to gift cigarettes in strong relationship contexts, and 66% were willing to do so in weak relationship contexts. Similarly, 54% expected to receive cigarettes in strong relationship contexts, and 55% expected to receive them in weak relationship contexts. The mean age of respondents was 39.5 years (SD=11.9), and 62% were male. Urban and rural residents were nearly balanced (47% rural, 53% urban). The mean household income was 108500 RMB (SD=137100), and 70% of participants were married or cohabiting, with 73% having at least one child. Education level was relatively high, with a mean of 3.82 (SD=1.33) on a 5-point scale (1=primary school to 5=postgraduate). Respondents’ pre-test perception of smoking hazards averaged 3.93 (SD=1.03), indicating general awareness of smoking-related harms.

**Table 1:** Descriptive characteristics of participants and group comparisons, China, June 2024 (N=744)

*Variables*	*Overall* *sample* *Mean (SD)*	*Group 1* *Mean (SD)*	*Group 2* *Mean (SD)*	*Group 3* *Mean (SD)*	*t* *Groups* *1 and 2*	*t* *Groups* *1 and 3*
Willingness to gift cigarettes in strong relationships	0.59 (0.58)	0.57 (0.50)	0.66 (0.48)	0.52 (0.50)	-1.87	1.12
Willingness to gift cigarettes in weak relationships	0.66 (0.48)	0.70 (0.46)	0.72 (0.45)	0.56 (0.50)	-0.49	3.04**
Expectation to receive cigarettes as gifts in strong relationships	0.54 (0.50)	0.51 (0.50)	0.60 (0.49)	0.49 (0.50)	-1.95	0.59
Expectation to receive cigarettes as gifts in weak relationships	0.55 (0.50)	0.56 (0.50)	0.61 (0.49)	0.48 (0.50)	-1.27	1.64
Age (years)	39.51 (11.93)	40.02 (11.58)	38.19 (11.97)	40.55 (12.03)		
Gender	0.62 (0.49)	0.58 (0.50)	0.62 (0.49)	0.64 (0.48)		
Urban/rural	0.47 (0.50)	0.50 (0.50)	0.51 (0.50)	0.39 (0.48)		
Income (RMB)	10.85 (13.71)	11.49 (17.40)	9.94 (9.12)	11.36 (14.79)		
Marital status	0.70 (0.46)	0.72 (0.45)	0.68 (0.47)	0.70 (0.46)		
Children	0.73 (0.45)	0.74 (0.44)	0.71 (0.46)	0.74 (0.44)		
Education level	3.82 (1.33)	3.87 (1.24)	3.90 (1.39)	3.70 (1.31)		
Smokers in the social circle	3.21 (0.94)	3.34 (0.91)	3.18(0.95)	3.16 (0.94)		
Change in perception of smoking hazards	0.04 (0.70)	0.07 (0.53)	0.06 (0.86)	0.11 (0.60)		
Post-test perception of smoking hazards	3.96 (1.06)	4.21 (0.93)	3.75 (1.16)	4.02 (1.00)		
Pre-test perception of smoking hazards	3.93 (1.03)	4.14 (0.93)	3.81 (1.11)	3.91 (0.99)		
Total, n	744	189	285	270	474	459

For binary categorical variables coded as 0/1, mean values correspond to proportions. For ordinal categorical variables (e.g. education level coded from 1=primary school to 5=postgraduate), mean values represent the average of coded categories. Group differences were assessed using independent-samples t-tests for consistency with the coding scheme.

***p≤0.001,

** p≤0.01,

*p≤ 0.05.

As shown in Table 2, respondents also exhibited differences in brand preferences. In strong relationship contexts, the mean gift brand tier was 1.91 (SD=0.69), significantly lower than in weak relationship contexts (mean=2.32, SD=0.66). Likewise, the expected gift tier was higher in weak relationships (mean=2.15, SD=0.68) than in strong relationships (mean=1.99, SD=0.70).

**Table 2:** Effects of interventions on willingness to gift and receive cigarettes in strong and weak relationships, China, June 2024 (N=744)

*Variables*	*Overall sample* *Mean (SD) n*	*Group 1* *Mean (SD) n*	*Group 2* *Mean (SD) n*	*Group 3* *Mean (SD) n*
Willingness to gift cigarettes in strong relationships	1.91 (0.69) 435	1.99 (0.69) 108	1.80 (0.69) 187	1.99 (0.69) 140
Willingness to gift cigarettes in weak relationships	2.32 (0.66) 552	2.45 (0.63) 132	2.24 (0.64) 234	2.34 (0.69) 186
Expectation to receive cigarettes as gifts in strong relationships	1.99 (0.70) 400	2.03 (0.65) 97	1.90 (0.71) 172	2.08 (0.71) 131
Expectation to receive cigarettes as gifts in weak relationships	2.15 (0.68) 409	2.30 (0.66) 105	2.01 (0.67) 175	2.21 (0.68) 129

Independent-samples t-tests (Table 1) comparing the control and intervention groups revealed no significant differences in most dependent variables. A slight difference was observed between the image warning group and the control group in willingness to gift cigarettes in weak relationships, but this difference was not statistically significant (p>0.05). Overall, baseline comparability suggests that neither the price nor the image intervention groups differed significantly from the control group in participants’ demographic characteristics or outcome measures.

### Regression results analysis

Compared with the control group (Tables 3–5), the price treatment group showed no significant difference in willingness to gift cigarettes in weak relationships (β= -0.19; 95% CI: -0.65–0.27). In weak relationship gifting, the effect of price is not significant (β= -0.19; 95% CI: -0.65–0.27), and similarly, no significant effect is observed in weak relationship receiving (β= -0.13; 95% CI: -0.58–0.32). For continuous covariates such as age and income, β coefficients correspond to a one-year increase in age and a one-unit increase in log-transformed household income, respectively.

**Table 3:** Ordered logistic regression analysis of willingness to gift cigarettes, and expectation to receive cigarettes, China, June 2024 (N=744)

	*Willingness to gift cigarettes*				*Expectation to receive cigarettes*			
	*Price treatment*		*Image warning treatment*		*Price treatment*		*Image warning treatment*	
	*Strong* *relationship* *gifting* *β (95% CI)*	*Weak relationship* *gifting* *β (95% CI)*	*Strong* *relationship* *gifting* *β (95% CI)*	*Weak relationship* *gifting* *β (95% CI)*	*Strong* *relationship gift* *receiving* *β (95% CI)*	*Weak relationship* *gift receiving* *β (95% CI)*	*Strong* *relationship gift* *receiving* *β (95% CI)*	*Weak relationship* *gift receiving* *β (95% CI)*
**Treatment**								
Price	0.13 (-0.31–0.58)	-0.19 (-0.65–0.27)	-	-	0.02 (-0.43–0.47)	-0.13 (-0.58–0.32)	-	-
Image	-	-	-0.39 (-0.83–0.04)	-0.88*** (-1.33 – -0.42)	-	-	-0.34 (-0.80–0.12)	-0.62** (-1.08 – -0.16)
**Control variables**								
Age (years)	-0.04*** (-0.07 – -0.02)	-0.32* (-0.06 – -0.01)	-0.03* (-0.06–0.01)	-0.04** (-0.06 – -0.01)	-0.05*** (-0.08 – -0.02)	-0.06*** (-0.09 – -0.03)	-0.03* (-0.05–0.00)	-0.04** (-0.07 – -0.01)
Gender (Male) (ref: Female)	0.66** (0.21–1.12)	0.66** (0.20–1.11)	0.99*** (0.53–1.46)	0.91*** (0.43–1.38)	1.18*** (0.73–1.64)	1.17*** (0.71–1.62)	1.34*** (0.85–1.84)	1.25*** (0.76–1.74)
Education level (Primary=1, Postgraduate=5, treated as ordinal)	0.01 (-0.20–0.21)	-0.20 (-0.41–0.00)	-0.02 (-0.21–0.17)	-0.13 (-0.32–0.06)	-0.16 (-0.37–0.05)	-0.19 (-0.40–0.02)	-0.13 (-0.33–0.07)	-0.17 (-0.37–0.03)
Urban/Rural (ref: Rural)	-0.13 (-0.57–0.32)	-0.02 (-0.46–0.43)	0.20 (-0.25–0.64)	0.50* (0.04–0.95)	-0.00 (-0.45–0.45)	0.04 (-0.40–0.49)	0.52* (0.06–0.99)	0.62** (0.15–1.08)
Income^§^	0.54*** (0.29–0.80)	0.43*** (0.18–0.69)	0.17 (-0.06–0.39)	0.06 (-0.17–0.28)	0.37** (0.12–0.63)	0.31* (0.06–0.56)	0.07 (-0.16–0.30)	0.01 (-0.22–0.24)
Marital status (Married/cohabiting) (ref: Single/divorced/widowed)	0.13 (-0.55–0.83)	0.48 (-0.22–1.18)	-0.22 (-0.94–0.49)	-0.10 (-0.82–0.62)	0.25 (-0.46–0.95)	0.54 (-0.16–1.24)	0.26 (-0.48–1.00)	0.23 (-0.51–0.97)
Children (Yes) (ref: No)	0.17 (-0.66–1.01)	-0.63 (-1.48–0.21)	0.74 (-0.11–1.60)	0.53 (-0.34–1.40)	0.15 (-0.68–0.98)	0.14 (-0.68–0.97)	-0.06 (-0.94–0.81)	0.34 (-0.54–1.21)
Smokers in the social circle	0.44*** (0.20–0.68)	0.25* (0.01–0.49)	0.36** (0.12–0.60)	0.14 (-0.10–0.37)	0.28* (0.04–0.52)	0.25* (0.01–0.49)	0.32* (0.07–0.58)	0.20 (-0.05–0.45)
Pre-test perception of smoking hazards	-0.66*** (-0.91 – -0.40)	-0.56*** (-0.82 – -0.30)	-0.78*** (-1.05 – -0.50)	-0.77*** (-1.06 – -0.48)	-0.87*** (-1.13 – -0.61)	-0.80*** (-1.06 – -0.54)	-0.97*** (-1.25 – -0.68)	-0.99*** (-1.28 – -0.70)
Change in perception of smoking hazards	-0.47** (-0.81 – -0.13)	-0.27 (-0.62–0.07)	-0.72*** (-1.12 – -0.32)	-0.81*** (-1.23 – -0.39)	-0.69*** (-1.03 – -0.35)	-0.62*** (-0.96 – -0.29)	-0.91*** (-1.32 – -0.50)	-0.95*** (-1.37 – -0.53)
Pseudo R^2^	0.19	0.13	0.18	0.17	0.23	0.21	0.24	0.24
Total, n	474	474	459	459	474	474	459	459

Dependent variables: willingness to gift cigarettes and expectation to receive cigarettes, each measured on a five-point Likert scale (1=very unwilling, 2=unwilling, 3=neutral, 4=willing, 5=very willing). The treatment variables of price and packaging come from the group assignments of the respondents: 1=control group, 2=price treatment group, 3=graphic warning treatment group.

§ To adjust the distribution of the income variable, the income variable in this regression analysis is log-transformed. β coefficients from ordered logistic regression, negative values indicate lower odds of selecting higher categories.

*** p≤0.001,

** p≤0.01,

* p≤0.05

**Table 4:** Ordered logistic regression results for brand tier preferences in receiving contexts, China, June 2024 (N=744)

	*Brand tier preference for gifting*				*Brand tier preference in gift receiving*			
	*Price treatment*		*Image warning treatment*		*Price treatment*		*Image warning treatment*	
	*Strong* *relationship* *gifting* *β (95% CI)*	*Weak relationship* *gifting* *β (95% CI)*	*Strong* *relationship* *gifting* *β (95% CI)*	*Weak relationship* *gifting* *β (95% CI)*	*Strong* *relationship gift* *receiving* *β (95% CI)*	*Weak relationship* *gift receiving* *β (95% CI)*	*Strong* *relationship gift* *receiving* *β (95% CI)*	*Weak relationship* *gift receiving* *β (95% CI)*
**Treatment**								
Price	-0.62** (-1.09–0.15)	-0.67** (-1.11–0.23)	-	-	-0.41 (-0.90–0.09)	-0.88*** (-1.38 – -0.39)	-	-
Image	-	-	0.06 (-0.44–0.56)	-0.13 (-0.59–0.32)	-	-	0.32 (-0.21–0.85)	-0.09 (-0.61–0.43)
**Control variables**								
Age (years)	-0.02 (-0.05–0.01)	0.01** (-0.02–0.03)	0.01 (-0.02–0.03)	0.01 (-0.02–0.04)	-0.01 (-0.03–0.02)	0.00 (-0.03–0.03)	0.01 (-0.02–0.04)	0.02 (-0.01–0.05)
Gender (Male) (ref: Female)	0.25 (-0.26–0.75)	0.39 (-0.06–0.84)	0.20 (-0.42–0.82)	0.29 (-0.24–0.82)	-0.35 (-0.89–0.20)	0.04 (-0.48–0.57)	-0.19 (-0.87–0.50)	-0.21 (-0.89–0.46)
Education level (Primary=1, Postgraduate=5, treated as ordinal)	-0.06 (-0.26–0.15)	0.24* (0.05–0.43)	0.11 (-0.11–0.34)	0.14 (-0.07–0.34)	-0.00 (-0.21–0.21)	0.07 (-0.14–0.27)	0.16 (-0.07–0.39)	0.21 (-0.02–0.44)
Urban/Rural (ref: Rural)	0.12 (-0.35–0.59)	0.46* (0.03–0.90)	0.31 (-0.21–0.83)	0.15 (-0.31–0.62)	0.07 (-0.42–0.56)	0.35 (-0.13–0.83)	0.54* (-0.00–1.08)	0.41 (-0.12–0.94)
Income^§^	0.29* (0.03–0.55)	0.40** (0.15–0.65)	0.30* (0.04–0.56)	0.48*** (0.25–0.72)	0.25 (-0.02–0.51)	0.21 (-0.06–0.47)	0.20 (-0.06–0.45)	0.24 (-0.02–0.49)
Marital status (Married/cohabiting) (ref: Single/divorced/widowed)	-0.52 (-1.30–0.25)	-0.13 (-0.84–0.58)	-0.63 (-1.40–0.14)	0.09 (-0.64–0.83)	-0.89* (-1.67–-0.11)	-0.52 (-1.33–0.29)	-0.09 (-0.88–0.70)	-0.20 (-0.99–0.58)
Children (Yes) (ref: No)	-0.13 (-1.04–0.77)	-0.45 (-1.30–0.39)	0.14 (-0.76–1.05)	-0.49 (-1.37–0.39)	-0.15 (-1.05–0.75)	-0.38 (-1.31–0.56)	0.01 (-0.92–0.93)	-0.29 (-1.23–0.66)
Smokers in the social circle	0.02 (-0.24–0.27)	0.13 (-0.12–0.37)	0.21 (-0.09–0.51)	0.33* (0.06–0.61)	0.29* (0.02–0.57)	0.28* (0.01–0.55)	0.47** (0.15–0.78)	0.44** (0.13–0.75)
Pre-test perception of smoking hazards	-0.08 (-0.32–0.16)	0.17 (-0.05–0.40)	0.12 (-0.16–0.39)	0.34* (0.07–0.61)	0.03 (-0.22–0.28)	-0.03 (-0.27–0.22)	0.19 (-0.11–0.49)	0.18 (-0.11–0.47)
Change in perception of smoking hazards	0.06 (-0.27–0.39)	-0.01 (-0.28–0.26)	0.34 (-0.11–0.78)	0.20 (-0.18–0.59)	0.11 (-0.19–0.41)	-0.15 (-0.47–0.17)	0.52* (0.06–0.99)	0.17 (-0.30–0.64)
Pseudo R^2^	0.04	0.08	0.04	0.07	0.05	0.06	0.06	0.05
Total, n	295	366	248	318	269	280	228	234

Dependent variable: brand tier (1=low-tier [Yuxi], 2=mid-tier [Zhonghua], 3=high-tier [Huanghelou 1916]).

§ To adjust the distribution of the income variable, the income variable in this regression analysis is log-transformed. β coefficients from ordered logistic regression, negative values indicate lower odds of selecting higher categories.

*** p≤0.001,

** p≤0.01,

* p≤0.05.

**Table 5:** Effects of interventions on changes in smoking hazard perception (China, June 2024– N=744)

	*Price Group smoking risk* *perception (post-test)* *β (95% CI)*	*Image Group smoking risk* *perception (post-test)* *β (95% CI)*
**Treatment**		
Price	-0.46* (-0.86 – -0.06)	
Image		0.08 (-0.38–0.54)
**Control variables**		
Age (years)	0.01 (-0.01–0.03)	0.00 (-0.03–0.03)
Gender (Male) (ref: Female)	-0.35(-0.77–0.08)	-0.87*** (-1.40 – -0.34)
Education level (Primary=1, Postgraduate=5, treated as ordinal)	0.02 (-0.16–0.20)	-0.05 (-0.26–0.15)
Urban/Rural (ref: Rural)	0.17 (-0.23–0.57)	-0.12 (-0.60–0.35)
Income^§^	-0.15 (-0.37–0.06)	-0.09 (-0.32–0.14)
Marital status (Married/cohabiting) (ref: Single/divorced/widowed)	0.23 (-0.40–0.86)	0.38 (-0.38–1.14)
Children (Yes) (ref: No)	-0.33 (-1.09–0.43)	-0.53 (-1.47–0.40)
Smokers in the social circle	0.07 (-0.14–0.29)	0.00 (-0.26–0.26)
Pre-test perception of smoking hazards	2.41*** (2.11–2.71)	3.00*** (2.63–3.38)
Pseudo R^2^	0.36	0.46
Total, n	474	459

Dependent variable: post-test perception of smoking risks measured on a 5-point Likert scale (1=very small, 2=small, 3=moderate, 4=large, 5=very large).

§ To adjust the distribution of the income variable, the income variable in this regression analysis is log-transformed. β coefficients from ordered logistic regression, negative values indicate lower odds of selecting higher categories.

*** p≤0.001,

**p≤0.01,

* p≤0.05.

Ordered logistic regression (Table 3) showed that, compared with the control group, the image warning group reported lower willingness to gift cigarettes in weak relationship contexts (β= -0.88; 95% CI: -1.33 – -0.42, p<0.001) and lower expectation to receive cigarettes (β= -0.62; 95% CI: -1.08 – -0.16, p<0.001). In these models, negative coefficients indicate lower odds of selecting higher categories on the 5-point Likert scale (1=very unwilling to 5=very willing). However, the effects are not significant in strong relationships (β= -0.39; 95% CI: -0.83–0.04 for giving and β= -0.34; 95% CI: -0.80–0.12 for receiving).

Controlling for covariates, the study finds that younger respondents (β= -0.04; 95% CI: -0.07 – -0.02, p≤0.001, for strong relationship giving), males (β=0.66; 95% CI: 0.21–1.12, p≤0.01), and those with higher income (β=0.54; 95% CI: 0.29–0.80, p≤0.001) are more likely to choose cigarettes as gifts. A greater number of smokers in one’s social circle (β=0.44; 95% CI: 0.20–0.68, p≤0.001) and lower perception of smoking harm (β= -0.66; 95% CI: -0.91 – -0.40, p≤0.001) are also positively associated with gifting behavior.

With respect to cigarette brand preferences, ordered logistic regression models (1=low-tier, 2=mid-tier, and 3=high-tier) showed that, compared with the control group, the price treatment group reported lower brand tier preferences in both strong (β= -0.62; 95% CI: -1.09 – -0.15, p≤0.01) and weak (β= -0.67; 95% CI: -1.11– -0.23, p≤0.01) relationship gifting contexts. Here, negative β coefficients indicate a greater likelihood of choosing lower tier rather than higher tier brands. In contrast, no significant differences in brand tier preference were observed for the image warning group relative to the control group, either in strong (β=0.06; 95% CI: -0.44–0.56) or weak (β= -0.13; 95% CI: -0.59–0.32) relationship contexts.

The results (Table 5) also show that graphic warning labels do not significantly increase perceived risk (β=0.08; 95% CI: -0.38–0.54), whereas price regulation unexpectedly reduces perceived risk (β= -0.46; 95% CI: -0.86 – -0.06, p≤0.05).

Pre-test and post-test perceptions of smoking risks were positively associated. For example, in the image warning group, each one-unit increase in pre-test perception was associated with higher post-test perception (β=3.00; 95% CI: 2.63–3.38, p≤0.001).

## DISCUSSION

This study employs survey experiments and data analysis to explore how graphic warning labels and price regulation reshape the social value of cigarettes. Firstly, graphic warning labels significantly reduce the social function of cigarettes in weak relationships. Specifically, after the addition of graphic warnings, respondents’ willingness to gift cigarettes in weak relationships decreases notably. This indicates that graphic warnings weaken the symbolic function of cigarettes as ‘social currency’ by altering their symbolic meaning rather than indirectly influencing behavior through heightened perceptions of smoking-related harm. Indeed, the results show that risk perception did not significantly increase following exposure to warning images, indicating that risk perception did not mediate the effect of graphic warnings on gifting behavior. This finding supports Reitzes et al.^19^ who argue that cigarette consumption is shaped not only by behavioral choice but also by social contexts and symbolic interactions. Secondly, the study finds that price regulation has a limited effect on the use of cigarettes as gifts. Even when prices are doubled, respondents’ willingness to gift cigarettes in strong relationships does not significantly decline, while in weak relationships, the change is minimal. Furthermore, price increases lower respondents’ perceptions of smoking-related harm, which may stem from the prevalent ‘price-quality effect’ in China, where higher priced products are often perceived as better quality and less harmful^20,21^. This finding underscores the limitations of price regulation in changing consumer behavior and suggests that policymakers should consider more diverse intervention strategies. As Chaloupka et al.^13^ note, while price increases can reduce smoking rates in some contexts, their impact is often limited when the perceived value of the product, including its social meaning, remains unchanged. This aligns with our findings that price increases have little effect on cigarette gifting behavior in close relationships, where the symbolic value of cigarettes as social gifts is less influenced by economic changes. Thirdly, the study confirms the ‘diminishing marginal value’ of social currency. In strong relationships, cigarettes’ symbolic meaning primarily supports emotional bonds and remains stable, whereas in weak relationships, the meaning relies heavily on external attributes (like packaging and price), making them more vulnerable to interventions such as graphic warnings. This rule offers a new theoretical perspective for understanding the mechanisms of cigarettes’ social functions and provides targeted guidance for formulating tobacco control strategies.

Finally, from a cultural perspective, the use of cigarettes as gifts is deeply ingrained in China’s tradition of reciprocity. Their symbolic meaning goes beyond economic value to serve as a vessel for cultural and social relationships. Therefore, single economic measures or health communication strategies may not effectively diminish the social function of cigarettes. This study suggests that future tobacco control campaigns should emphasize the symbolic attributes of cigarettes, using stronger graphic warnings or innovative designs to alter public perceptions and symbolic interpretations for long-term effectiveness^22^. As Hall et al.^23^ and Wu et al.^6^ point out , altering the symbolic meaning of cigarettes through packaging design and stronger health warnings is more likely to shift public perception and reduce their perceived value in social interactions.

### Limitations

This study has several limitations. First, although participants were randomly assigned to intervention groups, the randomization procedure was relatively simple and may not fully eliminate allocation bias. Blinding was not feasible because participants could clearly see group-specific materials. Second, although we adjusted for a number of demographic and behavioral covariates, the possibility of residual confounding cannot be excluded. Third, the outcomes relied on self-reported data, which may be subject to information bias or misclassification. Fourth, the sample was drawn from a specific population of students and social contacts, which may limit the generalizability of the findings to broader populations. Fifth, the study focused on general health persuasion through price increases and warning images but did not examine the effects of risk-specific warning labels (e.g. oral diseases, skin aging) that may have different impacts across demographic groups. Finally, the R² values of our models are relatively low, which is common in social science research focusing on behavioral outcomes, and this limits the overall explanatory power of the models.

## CONCLUSIONS

This study examined how cigarette packaging and pricing influence social gifting behaviors from the perspective of symbolic interactionism. The findings suggest that graphic warnings can reduce willingness to gift or receive cigarettes, while price increases may shift brand preferences toward lower tier products. These results provide theoretical and practical insights into the symbolic role of tobacco in gift-giving practices and may inform the development of public health strategies. However, further research with diverse populations and more specific health warning messages is needed to provide sufficient evidence for long-term policy recommendations.

## Supplementary Material



## Data Availability

The data supporting this research are available from the authors on reasonable request.
